# Impact of Different Grilling Temperatures on the Volatile Profile of Beef

**DOI:** 10.3390/foods14244239

**Published:** 2025-12-10

**Authors:** Fathi Morsli, Aidan P. Moloney, Frank J. Monahan, Peter G. Dunne, David T. Mannion, Iwona Skibinska, Kieran N. Kilcawley

**Affiliations:** 1 Food Quality and Sensory Science, Teagasc Food Research Centre, Moorepark, Fermoy, P61 C996 Co. Cork, Ireland; fathi.m.morsli@teagasc.ie (F.M.); david.mannion@teagasc.ie (D.T.M.); iwona.skibinska@teagasc.ie (I.S.); 2 UCD School of Agriculture and Food Science, University College Dublin, Belfield, D04 V1W8 Dublin, Irelandfrank.monahan@ucd.ie (F.J.M.); peter.dunne@ucd.ie (P.G.D.); 3 Teagasc, Animal & Grassland Research and Innovation Centre, Grange, Dunsany, C15 PW93 Co. Meath, Ireland; 4 School of Food and Nutritional Science, University College Cork, T12 R220 Cork, Ireland; 5 School of Food and Environmental Health, Technical University of Dublin, D07 EWV4 Dublin, Ireland

**Keywords:** beef, degree(s) of doneness, direct immersion-high capacity sorptive extraction, gas chromatography–mass spectrometry

## Abstract

The volatile profiles of beef steaks (*Longissimus lumborum*) were analysed both raw and grilled to five different internal temperatures, 55 °C, 60 °C, 71 °C, 77 °C, and 85 °C, representing very-rare, rare, medium rare, well-done, and very well-done, respectively. Volatile organic compounds (VOCs) were extracted using direct immersion high-capacity sorptive extraction (DI-HiSorb) and analysed by gas chromatography–mass spectrometry (GC–MS). A total of ninety-one VOCs were detected with forty-two significantly impacted by the degree of doneness, thirty of which had Variable in Projection score > 1. Principal Component Analysis produced three distinct clusters, (i) raw, (ii) very-rare, rare, and medium-rare, (iii) and well-done and very well-done. Direct immersion high-capacity sorptive extraction (DI-HiSorb) provided a comprehensive volatile profile of grilled beef steak across different degrees of doneness and revealed that the abundance of methyl esters. The main findings were that in relation to the degree of doneness methyl esters were significantly reduced, with both aldehydes and pyrazines increasing due to thermal lipid oxidation, Strecker degradation, and Maillard reaction, highlighting the significance of internal temperature on the volatile profile of steak during grilling.

## 1. Introduction

Traditionally, discussions on beef palatability have focused mainly on tenderness; however, beef flavour has gained increasing attention due to its complexity and significant influence on consumer preferences [1,2]. Beef flavour is composed of multiple sensory attributes, including taste, aroma, chemesthetic factors, and aftertaste [3]. Raw beef has little or no flavour apart from a blood-like taste, with flavour primarily developing during the cooking process [4,5,6,7]. Thus, the method of cooking has a significant impact on the resulting flavour profile. It is believed that the volatile organic compounds (VOCs) formed during cooking determine the aroma attributes and contribute significantly to flavour and therefore its sensory perception [6]. One of the most common methods of cooking is grilling, and previous studies have shown that grilling produces a complex and diverse aroma profile consisting of aldehydes, alkanes, ketones, alcohols, thiols, sulphur compounds, esters, amines, furans, lactones, and pyrazines [8,9,10,11]. These compounds are mainly formed through lipid thermal oxidation, the Maillard reaction, and Strecker degradation, which are the key chemical pathways that are promoted during grilling [8,9,10,12]. Together, these reactions generate a wide range of aroma-active compounds that contribute to the distinctive and appealing flavour of grilled beef. The internal cooking temperature directly affects the progress of these chemical reactions, and consequently, the volatile profile. As the internal temperature or the degree of doneness increases, Maillard, Strecker degradation and lipid thermal oxidation reactions intensify, altering the volatile profile of cooked beef [13,14].

Headspace solid-phase microextraction (HS-SPME) has been the most widely used procedure for volatile compound extraction post gas chromatography–mass spectrometry (GCMS) analysis in beef studies to date. However, a recent study demonstrated that direct immersion high capacity sorptive (DI-HiSorb) extraction GCMS is a promising technique for extracting volatile compounds from both raw and grilled beef [15]. The developed extraction technique effectively extracted a broad spectrum of volatile and semi-volatile compounds in both raw and grilled steak, notably higher molecular weight and less volatile esters, aldehydes, and acids that are not as easily extracted using headspace techniques [15].

To advance our understanding of beef flavour development, it is essential to examine how internal cooking temperatures shape the formation of VOCs. Although previous research has compared the volatile profiles of beef prepared by various cooking methods (e.g., boiling, roasting, and grilling) [14,16,17,18], few studies have investigated how different internal grilling temperatures influence the types and levels of specific volatiles. In this study, raw beef was included as a control to establish a baseline profile and clarify how heat during grilling alters VOC composition. The overall aim is to characterise VOCs changes across increasing degrees of doneness—representing very-rare to very well-done—using a novel DI-HiSorb GC–MS method, providing deeper insight into how grilling temperature potentially affects VOCs.

## 2. Materials and Methods

### 2.1. Sample Preparation Experimental Protocol and Cooking Procedure

Eighteen striploin steaks (*Longissimus lumborum*), pre-cut to 2.54 cm from the same animal were used in this study. The animal (a steer) was typical of cattle produced from pasture-based systems in Ireland. It was humanely slaughtered at a licensed commercial abattoir using standard procedures in accordance with the Regulations (EC) No. 1099/2009 and No. 853/2004 [19,20]. Following slaughter, the carcass was chilled according to standard commercial practice and muscle was recovered and vacuum-packaged at 48 h post-mortem.

This study involved the analysis of both raw and grilled beef samples. Steaks were thawed overnight under refrigeration, after which subcutaneous external fat was trimmed with a knife and discarded. Raw steaks were grilled at five different internal temperatures: 55 °C, 60 °C, 71 °C, 77 °C, and 85 °C, representing very-rare, rare, medium-rare, well-done, and very well-done degrees of doneness, respectively. Sample treatments and the grilling procedures were performed according to optimized parameters previously established [15]. Analysis was carried out with three experimental and three analytical replicates, thus each experimental temperature (raw, 55 °C, 60 °C, 71 °C, 77 °C, and 85 °C) was carried out in triplicate, with volatile analysis undertaken in triplicate for each sample. Grilling was conducted according to the guidelines of the American Meat Science Association [21], with slight modifications based on Gardner and Legako (2018) and Kilgannon et al. (2020) [22,23]. A clamshell grill (Tefal Grill, Model G03-M, Group SEB UK Ltd., Datchet, UK) was preheated for 15 min to ensure a stable external surface temperature of 220 °C. Each steak was positioned at the centre of the grill, and both the upper and lower surfaces were grilled simultaneously. Internal temperatures were monitored using a probe thermometer (Sensor-Tech Ltd., Dublin, Ireland). Once the designed internal temperatures for each sample 55 °C, 60 °C, 71 °C, 77 °C, and 85 °C were achieved, steaks were removed from the grill using tongs and rested on aluminium foil for at least 2 min, before further analysis.

Throughout this study, raw and grilled beef samples are denoted as ‘R’ and ‘G’, respectively, for ease of reference. Samples ‘R’ represents raw beef, while samples ‘G55’, ‘G60’, ‘G71’, ‘G77’, and ‘G85’ represent the five different internal temperature levels: 55 °C, 60 °C, 71 °C, 77 °C, and 85 °C, respectively. The procedure below is a modification from Fathi et al., (2015) [15]. Fifty (50) g of chopped sample were mixed with 50 mL of ultrapure water in an inert 250 mL plastic container, placed on ice. The mixture was then homogenized using an Ultra-Turrax (IKA-Werke GmbH & Co., Staufen im Breisgau, Germany) with a probe at speed level 6 for 2 min for ‘R’ samples and 3 min for ‘G’ samples, resulting in a smooth, homogeneous paste. Two (2) g of the homogeneous paste were transferred into a 20 mL amber headspace vial (Element Lab Solutions, Maynooth, Ireland), followed by the addition of 13 mL of 15% methanol (MeOH). An internal standard solution consisting of 0.5 mL of 4-methyl-2-pentanol and 2-methyl-3-heptanone (50 mg L^−1^ in ultrapure water) was added to each vial. These internal standards were used to monitor the integrity of the extraction process. Vials were then sealed with specialized HiSorb septum caps (Markes International Ltd., Bridgend, UK). Each sample was extracted using a polydimethylsiloxane (PDMS) HiSorb probe (Markes International Ltd.) in a HiSorb Agitator (Markes International Ltd.).

### 2.2. Volatile Extraction and GC-MS Analysis

The procedure used was identical to that described by Fathi et al. (2025) [15].

### 2.3. Data Processing and Identification of VOCs

Analysis was undertaken using Mass Hunter Qualitative Analysis Software (version B.08.00) (Agilent Technologies Ltd., Santa Clara, CA, USA). Compound identification was performed by comparing mass spectra against NIST 2014 mass spectral library MS searching (v.2.3, Gaithersburg, MD, USA), and an in-house library created using authentic compounds with target and qualifier ions and linear retention indices for each compound using Kovats index. Spectral deconvolution was also performed to aid with identification of compounds using Automated Mass spectral Deconvolution and Identification System (AMDIS). Batch processing of samples was carried out using MetaMS [24]. MetaMS is an open-source pipeline for GC-MS-based untargeted metabolomics.

To monitor the performance of the GC-MS operating conditions, an external standard solution consisting of 1-butanol, dimethyl disulphide, butyl acetate and cyclohexanone (Merck Life Science Ltd., Arklow, Ireland) was analysed. A stock solution of these compounds was prepared at 1000 mg L^−1^ in methanol. For analysis by thermal desorption GC-MS, this solution was diluted to 10 mg L^−1^ using ultrapure water and 10 µL was added to a tenax TA/carbograph thermal desorption tube. A tube was analysed at the start and end of each series of samples. The peak areas of the external standards were monitored to ensure they were within a specified tolerance (10% coefficient of variation) to ensure that the instrumentation was performing within specification during the analysis.

### 2.4. Statistical Analysis

The data was collated in Excel (Microsoft Office, Redmond, WA, USA). The sensitivity, selectivity, and reproducibility of each sample was undertaken in three experimental and three analytical replicates and compared in terms of (i) the number of VOCs extracted, (ii) the total abundance equating to the total peak area of identified VOCs, (iii) and the % standard deviation of total peak area of identified VOCs. Histograms outlining the total abundance of peak areas of identified VOCs for each sample were created using Excel (Microsoft Office, Redmond, WA, USA). A one-way analysis of variance (ANOVA) was conducted on the total peak abundance using R (v.4.4.2, R Foundation for Statistical Computing, Vienna, Austria). Principal Component Analysis (PCA) was performed to visualize patterns related to each sample to assess the association of VOCs with different degrees of doneness. PCA analyses were conducted using the FactoMineR and factoextra packages in R (v.4.4.2, R Foundation for Statistical Computing, Vienna, Austria). Histograms outlining the percentage of chemical classes of each heat treatment were created using Excel (Microsoft Office, Redmond, WA, USA). One-way ANOVA was conducted on the abundance of chemical classes across all heat treatment to assess the effect of degree of doneness on chemical classes. In Metaboanalyst v6.0 (https://www.metaboanalyst.ca/, accessed on 2 March 2025) [25], data were scaled, the data was mean centred and divided by the standard deviation of each variable. After data normalization and processing, post hoc comparisons were performed using (Honestly Significant Difference) test to find the chemical classes most impacted by degree of doneness (*p* < 0.05), with false discovery rate adjustment applied to control for multiple comparisons. The impact of different degrees of doneness relating to each internal temperature on the volatile profile of beef was assessed using Variable Importance in Projectionon (VIP) analysis and one-way ANOVA. Both analyses were performed in MetaboAnalyst v6.0 (https://www.metaboanalyst.ca/, accessed on 2 March 2025) [25]. Data filtering was applied using the interquartile range method to remove variables with low variance (<5%) across the samples, which are unlikely to contribute meaningfully to downstream analyses. Missing values were imputed using the limit of detection method, which replaces values below the detection limit with one-fifth of the minimum observed positive value for each feature. The data were then scaled, by mean centring and dividing each variable by its standard deviation. After data processing and normalization, Partial Least Squares Discriminant Analysis (PLS DA) and one-way ANOVA were performed. For PLS DA, model performance was assessed by 5-fold cross-validation using Q2 as the performance metric, which is an estimate of the predictive ability of the model and is calculated via cross-validation. Interpretation of the PLS-DA model was based on the VIP, with any volatiles with a VIP score ≥ 1 considered key discriminators. For the one-way ANOVA, post hoc comparisons were performed using Tukey’s (Honestly Significant Difference) test to identify individual VOCs significantly impacted by degree of doneness (*p* < 0.05), with false discovery rate adjustment applied to control for multiple comparisons.

## 3. Results and Discussion

### Comparison of Volatile Organic Compounds (VOCs) and Chemical Classes in Raw and Grilled Beef as Impacted by Degree of Doneness

A total of ninety-one individual VOCs were identified across all samples (Table 1), consisting of the following chemical classes: aldehydes (22), esters (17), alkanes (15), acids (10), benzenes (7), ketones (6), alcohols (3), furans (3), pyrazines (3), lactones (2), alkene (1), amine, (1) and terpene (1).

**Figure 1:**
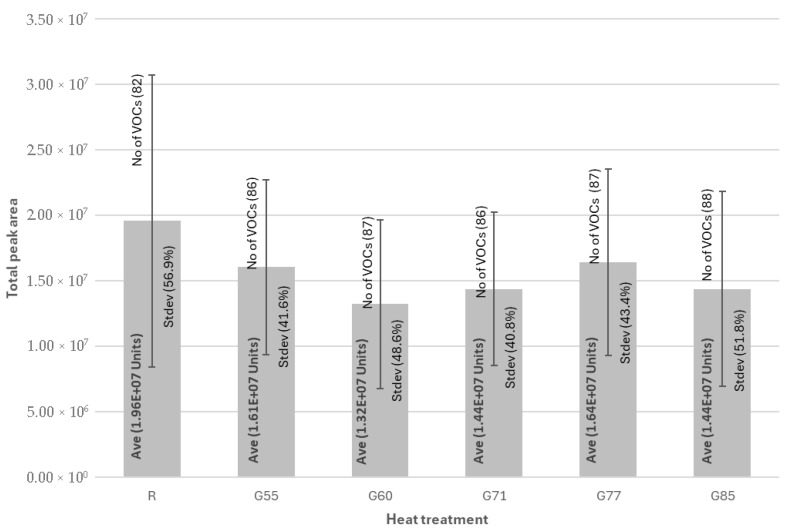
Total abundance of volatiles, standard deviation, and number of volatiles extracted in raw and grilled beef. No of VOCs: Number of individual VOC extracted by each technique. Ave: Average total peak area of identified volatiles expressed in arbitrary units. Stdev: Standard deviation of total peak area of identified volatiles, expressed as a percentage of the total. R (raw), G55 (very-rare), G60 (rare), G71 (medium-rare), G77 (well-done), and G85 (very well-done).

The number of VOCs increased due to thermal processing associated with increased internal temperature or degree of doneness, as highlighted in Figure 1. The greatest number of VOCs were identified in G85 (88) and the lowest in R (82). In contrast, the greatest total abundance (total peak area) was achieved in R (1.96 × 10^7^ units), while the lowest abundance was in G60 (1.32 × 10^7^ units). The loss of abundance from R is due to the high volume of esters in raw beef, many of which were degraded by hydrolysis due to the thermal treatment related to different degrees of doneness, also noted by Wang et al. (2017) [7]. There was no statistical difference in relation to the level of abundance which is part was due to high standard deviation which ranged from 40.8% (G71) to 56.9% (R). The high standard deviation is not uncommon in such analysis [26]. However, it is important to note that as beef is not a homogenous product, reproducibility indices in volatile extraction techniques will be higher than many other sample types despite the inclusion of a physical homogenisation step in the sample preparation. The viscosity of the emulsion is also likely influencing absorption onto the PDMS phase used in this study. Other sample-dependent variables—such as pH, salt concentration, and fat and protein composition—can also affect extraction behaviour and therefore impact reproducibility, particularly when using a direct-immersion (DI) approach [27]. In this study, probes were cleaned according to the manufacturer’s recommendations, and blanks were run between samples to monitor and minimize any carryover effects.

**Figure 2:**
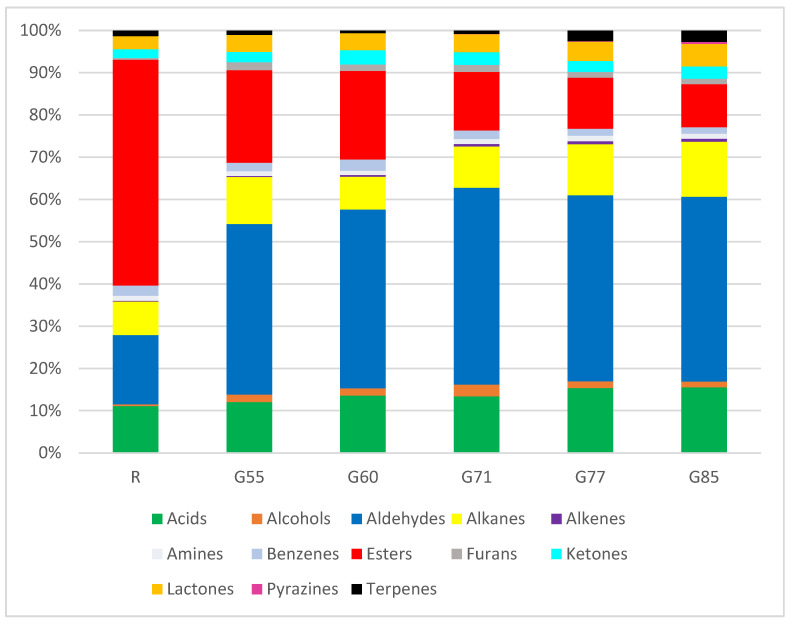
Chemical classes expressed as a percentage in raw and grilled beef. R (raw), G55 (very-rare), G60 (rare), G71 (medium-rare), G77 (well-done), and G85 (very well-done).

Figure 2 is a visual representation of the % of each chemical class in each sample. In relation to the chemical classes, alcohols, aldehydes, esters, furans and pyrazines were significantly impacted by degree of doneness (*p* < 0.01), as were terpenes (*p* 0.03) and alkenes (*p* 0.02) (Table S1). The chemical classes: acids, alkanes, amines, benzenes, ketones and lactones expressed on a percentage basis were not impacted by degree of doneness. It is immediately apparent how significant esters are in the R sample and their subsequent decrease on a percentage basis with increasing degree of doneness. The percentage abundance of esters decreased the most once heat-treatment (grilling) was applied, where the largest decrease occurred from R (53.5%) to G55 (21.9%). The high levels of esters in raw steaks are due to the alcohols and acids, which condense to form esters [7]. Samples grilled to higher degrees of doneness, G77 (12.1%) and G85 (10.2%), had the lowest levels of esters (Table S1). Other studies have also found a decrease in the abundance of esters post cooking [7,13,15,28,29]. Esters are formed through a condensation reaction between the hydroxyl group (-OH) of an alcohol and the carboxyl group (-COOH) of acid, with the elimination of water [30]. Thermal energy during grilling was likely responsible for the loss of esters with moisture changes also a possible factor [7,14,22,31]. The percentage of aldehydes increased rapidly from R (16.4%) to G55 (40.4%) and remained high in G60 (42.3%), G71 (46.6%), G77 (44.0%), and G85 (44.0%) (Table S1). Most of the aldehydes generated during grilling are derived from lipid oxidation, with some from Strecker degradation [9,10,13,22,29].

The percentage of alcohols immediately increased (*p* < 0.01) with the application of grilling, but decreased at the highest degrees of doneness; R (0.4%), G55 (1.8%), G60 (1.8%), G71 (2.7%), G77 (1.6%), and G85 (1.4%). Initial increases maybe due to hydrolysis of esters, but subsequent decreases may indicate degradation or losses due to volatility [14]. Pyrazines were absent until G71 (0.02%), and increased thereafter, G77 (0.1%) and G85 (0.5%), highlighting formation due to Maillard reactions at the higher degrees of doneness [32,33,34]. The percentage of furans increased from R (0.4%) to G55 (1.9%), with the application of heat during grilling, with levels varying thereafter; G60 (1.5%), G71 (1.7%), G77 (1.4%), and G85 (1.23%). In this study, with respect to the furans, most were primarily related to lipid oxidation. The percentage of terpenes (only one terpene was identified—neophytadiene), initially decreased from R (1.3%) to G55 (1.0%) with the application of grilling but subsequently increased at the higher degree of doneness; G77 (2.5%), G85 (2.7%). These changes with degree of doneness may indicate that neophytadiene is a product of lipid oxidation as suggested by Petron et al. (2005) that be maybe enhanced by thermal activity [35]. The percentage of alkenes (only one alkene identified—1-octene) also increased with degree of doneness (*p* 0.02), R (0.2%)–G85 (0.7%), which is also derived from lipid oxidation.

Of the ninety-one VOCs identified in these samples, forty-two were statistically different (*p* < 0.05) based on degree of doneness (Table 1). Twenty-two aldehydes were identified in total, with most derived from lipid oxidation, apart from 3-methyl butanal, 4-ethyl benzaldehyde, benzaldehyde, and benzeneacetaldehyde, which are Strecker-derived aldehydes. Fifteen aldehydes were found to be significantly impacted by the degree of doneness. Many of these were straight-chain aliphatic aldehydes; hexanal, heptanal, octanal, nonanal, decanal, dodecanal, tridecanal, and tetradecanal, and six branched chain aldehydes; (E,E)-2,4-heptadienal, (E)-2-octenal, (E)-2-nonenal, (E,E)-2,4-decadienal, 2,4-decadienal, and 2-undecenal. The most abundant aldehyde was nonanal; R (5.95%), G55 (9.95%), G60 (12.33%), G71 (12.62%), G77 (12.07%), and G85 (12.72%) and has previously been demonstrated to increase in beef with increasing cooking temperatures [13]. The only other aldehyde that was significantly different due to the degree of doneness, which was not derived from lipid oxidation, was benzaldehyde (*p* < 0.01); R (0.21%), G55 (0.35%), G60 (0.57%), G71 (0.51%), G77 (0.50%), and G85 (0.58%). Benzaldehyde is generated through Strecker degradation/Maillard reaction [22,29] and was previously found to increase in beef cooked at high internal temperatures (>85 °C) [13]. Many of these aldehydes are potentially important flavour-active VOCs in grilled beef [9,10], in part because of their relatively low odour thresholds. These aldehydes are thought in general to contribute `fruity’, `fatty’, and `flowery’ aromas to beef [36,37]. This result highlights the significance of lipid oxidation in relation to changes in aldehydes in relation to the degree of doneness.

The two most abundant esters in the R samples were methyl hexadecanoate (16.48%) and methyl tetradecanoate (10.1%), with methyl hexadecanoate remaining very abundant; G55 (10.75%), G60 (10.52%), G71 (7.87%), G77 (6.70%), and G85 (5.27%), see Table 1. Seventeen esters were identified, and all were significantly impacted by the degree of doneness (*p* < 0.01). Sixteen of the seventeen esters identified were methyl esters, with butyl isobutyrate (butyl-2-methylpropanoate) also detected. It is likely that methyl esters predominate in beef because mammalian tissues contain a variety of methylated biomolecules such as phospholipids that can carry methylated head groups, and by the fact that red muscle cells have active methyl transferases and S-adenosylmethionine cycles because of sustained oxidative metabolism that produce endogenous methanol, making it available for ester formation [38,39]. Methyl esters appear to be a less significant chemical class in other meats, for example, chicken which has less oxidative metabolism and a lower phospholipid content [40]. Methyl esters have been reported in many studies on beef [7,12,41,42,43]. Butyl isobutyrate like methyl-2-methylbutanoate were not detected in the R samples, but present at low abundances in the G55–G85 samples (butyl isobutyrate < 0.5%, and methyl-2-methylbutanoate < 1.0%) (Table 1). The generation of these two esters during cooking is likely related to the creation of the acids, 2-methylbutanoate and 2-methylpropanoate via Strecker degradation, that are substrates in their formation. Presumably, these acids are formed during the grilling process (even though these acids were not detected in this study) and are likely rapidly condensed to create the esters. Methyl octanoate, on the other hand, was present in R (2.48%), G55 (0.98%), and G60 (0.86%), but absent in G71 (0%), G77 (0%), and G85 (0%). As mentioned, most esters decreased with degree of doneness and the percentage decrease from R to G85 were as follows: methyl heptanoate (0.47–0.05%), methyl hexanoate (0.78–0.12%), methyl octanoate (2.48–0%), methyl 3-methylbutanoate (6.25–0%), methyl nonanoate (3.59–0.19%), methyl hexadecanoate (16.48–5.27%), methyl decanoate (4.25–0.37%), methyl tetradecanoate (10.01–0.79%), methyl palmitoleate (3.82–0.79%), methyl dodecanoate (1.25–0.08%), methyl pentadecanoate (1.93–0.29%), methyl myristoleate (1.82–0%), and methyl azelaaldehydate (0.04–0.04%). To our knowledge, this is the first study were methyl azelaaldehydate (methyl 9-oxononanoate) was detected in beef samples. It was previously detected in a meat-like systems [44] and maybe generated by thermal lipid oxidation during cooking by the cleavage of the C=C bond in hydroperoxides [45]. It may also be an intermediate compound as abundance decreased as degree of doneness increased. The proliferation of longer chain, less volatile esters identified in this study is mainly due to the choice of DI HiSorb as the extraction approach [15], as such esters are typically not identified by headspace methods due to their larger molecular weight.

Even though fifteen alkanes were identified, only three were significantly (*p* < 0.05) impacted by degree of doneness. Alkanes are lipid-derived volatiles [46]. Octane was only present in grilled beef and in general increased with degree of doneness; G55 (1.37%) to G77 (2.30%), and decreased at the highest internal grilling temperature G85 (1.62%). Domínguez et al. (2014) had also reported high levels of alkanes after grilling [29]. However, due to their high odour thresholds and odour attributes, alkanes are not considered important flavour compounds in meat [47,48]. All three pyrazines detected, 2,6-dimentylpyrazines, 3-ethyl-2,5-dimethyl pyrazine, and 2,5-dimethyl-3-isoamylpyrazine, were significantly impacted by the degree of doneness (*p* < 0.01). Pyrazines are products of the Maillard reaction [32,33,34] and were only identified at the higher grilling temperatures. 2,6-Dimethylpyrazine was only generated in G71 (0.02%), G77 (0.03%), and G85 (0.07%), highlighting that it increased with higher temperatures. 2,5-Dimethyl-3-isoamylpyrazine was only generated at G77 (0.12%) and G85 (0.30%), again increasing with the increase in grilling temperatures, and was also the most abundant pyrazine. 3-Ethyl-2,5—dimethyl-pyrazine was only generated at G85 (0.13%).

**Figure 3:**
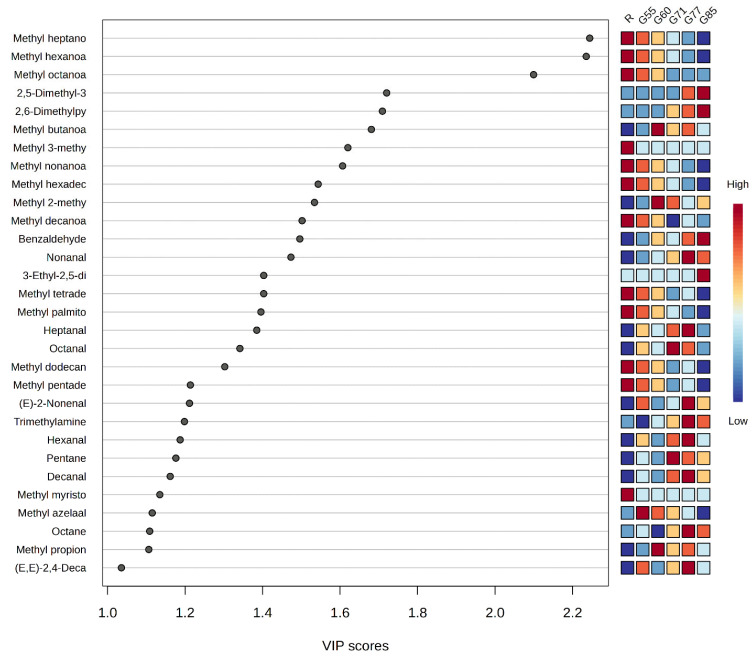
Variable Importance In Projection plot of individual volatile organic compounds most significantly impacted by different degrees of doneness. List of compounds (full names) in order of highest VIP score to the lowest: methyl heptanoate, methyl hexanoate, methyl octanoate, 2,5-dimethyl-3-isoamylpyrazine, 2,6-dimethylpyrazine, methyl butanoate, methyl 3-methylbutanoate, methyl nonanoate, methyl hexadecanoate, methyl 2-methylbutanoate, methyl decanoate, benzaldehyde, nonanal, 3-ethyl-2,5-dimethylpyrazine, methyl tetradecanoate, methyl palmitoleate, heptanal, octanal, methyl dodecanoate, methyl pentadecanoate, (E)-2-nonenal, trimethylamine, hexanal, pentane, decanal, methyl myristoleate, methyl azelaaldehydate, octane, methyl propionate, and (E,E)-2,4-decadienal, and R (raw), G55 (very-rare), G60 (rare), G71 (medium-rare), G77 (well-done), and G85 (very well-done).

Trimethylamine was the only amine detected and is characterised by a ‘fishy’ odour (Table 2). Trimethylamine was present in both raw and grilled beef and is generally considered as an indicator of bacterial storage [49]. However, it was significantly impacted by degree of doneness (*p* 0.02); R (1.18%), G55 (1.16%), G60 (1.01%), G71 (1.19%), G77 (1.30%), and G85 (1.19%), indicating formation/degradation as intermediate volatile dependent upon degree of doneness (Table 1). Only one (1-octanol) of the three alcohols identified was impacted by degree of doneness (*p* < 0.01). 1-Octanol is also a product of lipid oxidation, but may also be formed from the hydrolysis of esters [14]. Levels varied with degree of doneness; R (0.26%), G55 (1.29%), G60 (1.17%), G71 (1.22%), G77 (1.09%), and G85 (0.92%). Only three furans (2-furanmethanol, 2-pentylfuran, and 2-octylfuran) were identified in these samples with both 2-pentyl furan; R (0.28%), G55 (1.70%), G60 (1.33%), G71 (1.51%), G77 (1.19%), G85 (1.19%), and 2-octylfuran; R (0.02%), G55 (0.08%), G60 (0.06%, G71 (0.07%), G77 (0.07%), and G85 (0.08%), significantly impacted by degree of doneness (*p* < 0.01). Both are also products of lipid oxidation. None of the acids, benzenes, ketones, lactones, or the terpene and alkene detected were impacted by degree of doneness, highlighting that these VOCs have little or no role in sensory perception of grilled steak. Even though forty-two VOCs were found to be significantly altered by the degree of doneness, a VIP was undertaken to determine the most significantly impacted by the degree of doneness (Figure 3). This highlights that of the ninety-one VOCs identified thirty were significantly impacted by the degree of doneness, as they had a VIP > 1, of which sixteen were esters and six were aldehydes and two alkanes, and all were derived from lipid oxidation. There were also three pyrazines from Maillard reaction and one aldehyde from Strecker degradation, plus trimethylamine, where levels varied with degree of doneness (Table 2). These results highlight that degree of doneness has the greatest impact on mainly ester degradation, enhanced thermal lipid oxidation with some Strecker degradation, and only an impact of Maillard reactions at the highest internal grilling temperatures.

**Figure 4:**
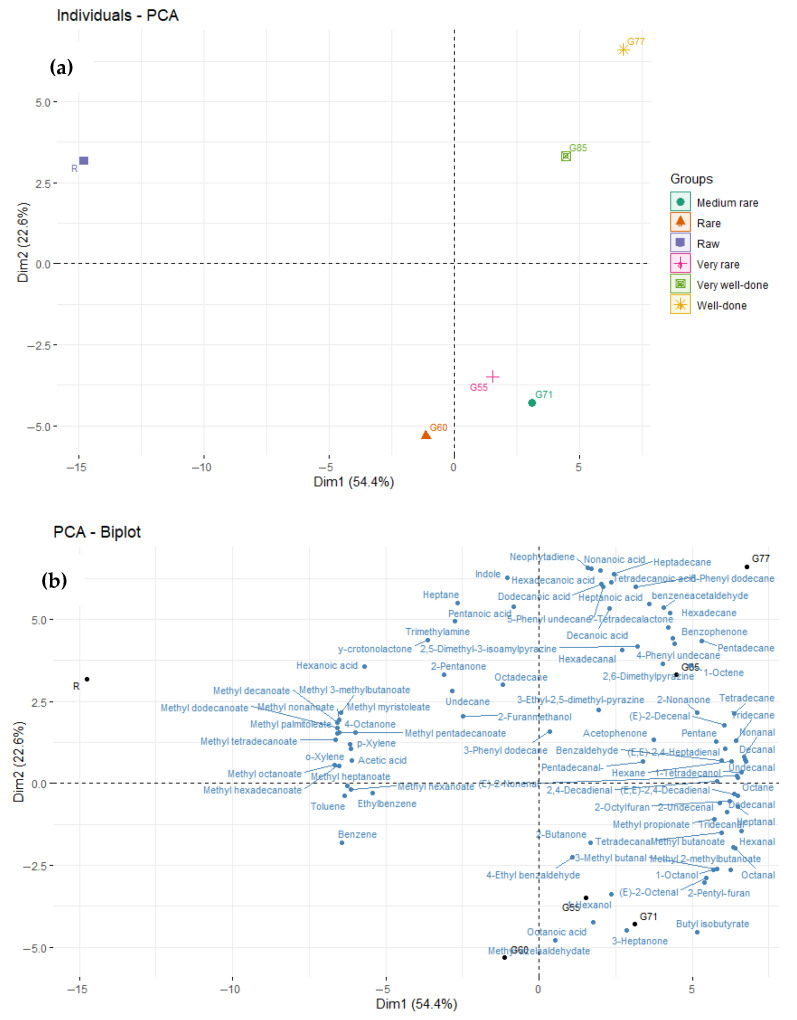
Principal Component Analysis of the discrimination between raw and grilled samples based on their volatile profile. (**a**) Sample discrimination, and (**b**) sample discrimination plus individual volatile components. R (raw), G55 (very-rare), G60 (rare), G71 (medium rare), G77 (well-done), and G85 (very well-done).

Figure 4a,b presents a PCA plot illustrating the separation between raw samples and different degrees of doneness based on their volatile profiles. The first dimension accounts for 54.4% of the total variation, while the second dimension explains 22.6%. The R samples are clearly isolated on the extreme negative side of the first dimension, whereas the very-rare (G55), rare (G60), and medium-rare (G71) samples visually cluster together on the negative side of the second dimension. It is evident that the R sample is most closely associated with a proliferation of methyl esters and that the VOC profiles of G55, G60, and G71 are somewhat similar and most strongly associated with 3-heptanone, octanoic acid, methyl azelaaldehydate, hexanol, and butyl isobutyrate among others. The application of higher internal grilling temperatures alters the VOCs profile as G77 (well-done) and G85 (very well-done) are visually grouped on the positive side of both dimensions, distinctly separated from the other two clusters, and visually grouped on the positive side of both dimensions. G77 and G85 are associated with the most complex range of VOCs, including the pyrazines, many alkanes, aldehydes, benzenes, lactones, and acids, as well as an alkene and a terpene. The pyrazines are likely a major factor in the position of G77 and G85 and are very important odour active compounds [53,54] contributing to ‘coffee’, ‘nutty’, ‘sweet’, and ‘fragrant’ aromas (Table 2).

This study has provided a deeper insight into how grilling temperature affects VOCs, with some very definitive changes once heating is applied, especially indicative of losses in methyl esters. It was also apparent that those samples grilled to internal temperatures of G55, G60, and G71 were more similar in terms of VOCs profile, mainly driven by thermal lipid oxidation, and this may indicate that any potential differences in sensory characteristics may be quite subtle in such samples. Those samples generated at the two highest internal grilling temperatures (G77 and G85), are quite distinct in comparison to all the other samples. These differences are mainly centred around the inclusion of pyrazines (Maillard reaction), an increase in benzaldehyde (Strecker degradation), and further losses of methyl esters (hydrolysis). Thus, such changes are likely to alter the sensory properties especially with the inclusion of the aromatic pyrazines. Again, both of these samples (G77 and G85) are quite similar in terms of volatile profile and therefore any perceived sensory differences may also be subtle. This study has also highlighted the benefits of DI-HiSorb in capturing a greater amount of VOCs, especially the more semi-volatile, such as the longer chain esters. However, it is worth reiterating that no single extraction method can provide an absolute volatile profile due to inherent bias. The main factors impacting the volatile profiles generated in this study are the use of DI and the choice of HiSorb phase (PDMS) and its capacity. The use of other volatile techniques and the incorporation of olfactometry and/or descriptive sensory science would likely provide much more definitive correlation between individual VOCs and sensory and aromatic perception.

## 4. Conclusions

This study characterised the volatile profile of beef steaks across increasing internal grilling temperatures using DI-HiSorb GC–MS. A total of ninety-one volatile compounds were identified, thirty of which were significantly influenced by degree of doneness. Higher internal temperatures (77–85 °C) produced distinct volatile patterns dominated by aldehydes and pyrazines formed through either lipid oxidation, Strecker degradation, and Maillard reactions, while methyl ester concentrations declined sharply with the application of heat. These findings confirm that internal temperature is a major driver of beef aroma chemistry and demonstrates the effectiveness of DI-HiSorb for capturing semi-volatile compounds often underestimated by traditional headspace methods. The results provide a more complete understanding of how thermal reactions shape beef flavour and can support the optimisation of cooking practices to enhance sensory quality. Future work should incorporate sensory evaluation and consumer studies to directly relate these chemical changes to flavour perception.

## Figures and Tables

**Table 1:** Volatile compounds extracted in raw and grilled beef.

Chemical Class	Individual VOCs	CAS Number	LRI	Ref LRI	Volatiles Expressed as Percentage of Total (%)						
					R	G55	G60	G71	G77	G85	*p* Value
Aldehydes	3-Methyl butanal	590-86-3	653	651	2.21	5.59	5.56	5.29	4.55	5.33	ns
	Hexanal	66-25-1	802	801	1.12	3.78	4.16	4.49	4.06	3.86	<0.01
	Heptanal	111-71-7	904	901	1.21	4.69	5.25	6.17	5.94	4.75	<0.01
	Benzaldehyde	100-52-7	970	960	0.21	0.35	0.57	0.51	0.50	0.58	<0.01
	Octanal	124-13-0	1005	1004	0.57	3.01	3.36	3.97	3.29	2.93	<0.01
	(E,E)-2,4-Heptadienal	4313-03-5	1015	1014	0.00	0.19	0.13	0.26	0.32	0.17	<0.01
	(E)-2-Octenal	2548-87-0	1061	1061	0.10	0.77	0.91	0.98	0.81	0.50	<0.01
	Nonanal	124-19-6	1108	1106	5.95	9.95	12.33	12.62	12.07	12.72	<0.01
	(E)-2-Nonenal	18829-56-6	1164	1160	0.00	0.71	0.48	0.58	0.76	0.64	<0.01
	4-Ethyl benzaldehyde	4748-78-1	1174	1164	0.43	0.68	0.63	0.76	0.62	0.39	ns
	Decanal	112-31-2	1209	1205	0.35	0.58	0.68	0.71	0.67	0.70	<0.01
	(E)-2-Decenal	3913-81-3	1267	1266	0.60	1.59	1.97	1.44	2.15	2.05	ns
	(E,E)-2,4-Decadienal	25152-84-5	1302	1299	0.00	0.33	0.22	0.31	0.36	0.26	<0.01
	Undecanal	112-44-7	1311	1309	0.12	0.23	0.24	0.25	0.24	0.27	ns
	2,4-Decadienal	2363-88-4	1326	1317	0.09	1.00	0.54	0.84	1.01	0.67	<0.01
	2-Undecenal	2463-77-6	1370	1369	0.31	1.89	1.11	1.61	1.60	1.51	<0.01
	Dodecanal	112-54-9	1413	1401	0.14	0.32	0.29	0.33	0.30	0.33	<0.01
	Tridecanal	10486-19-8	1516	1505	0.15	0.48	0.36	0.48	0.42	0.45	0.01
	Tetradecanal	124-25-4	1618	1615	0.24	0.80	0.61	0.82	0.66	0.75	0.02
	Pentadecanal	2765-11-9	1716	1707	0.88	1.45	1.07	1.51	1.27	1.37	ns
	Hexadecanal	629-80-1	1818	1819	1.69	1.98	1.79	2.58	2.33	3.46	ns
	benzeneacetaldehyde	122-78-1	1052	1051	0.05	0.07	0.08	0.08	0.11	0.12	ns
Esters	Methyl propionate	554-12-1	626	620	0.11	0.18	0.27	0.22	0.21	0.22	<0.01
	Methyl butanoate	623-42-7	720	719	0.18	0.59	0.84	0.73	0.66	0.71	<0.01
	Methyl 2-methylbutanoate	868-57-5	775	774	0.00	0.85	1.16	1.05	0.84	0.97	<0.01
	Methyl 3-methylbutanoate	556-24-1	776	774	6.25	0.00	0.00	0.00	0.00	0.00	<0.01
	Methyl hexanoate	106-70-7	923	922	0.78	0.61	0.42	0.22	0.18	0.12	<0.01
	Butyl isobutyrate	97-87-0	951	951	0.00	0.33	0.39	0.40	0.22	0.26	<0.01
	Methyl heptanoate	106-73-0	1023	1022	0.47	0.33	0.22	0.12	0.08	0.05	<0.01
	Methyl octanoate	111-11-5	1124	1126	2.48	0.98	0.86	0.00	0.00	0.00	<0.01
	Methyl nonanoate	1731-84-6	1223	1225	3.59	0.44	0.32	0.28	0.19	0.19	<0.01
	Methyl decanoate	110-42-9	1324	1322	4.25	0.85	0.86	0.18	0.41	0.37	<0.01
	Methyl azelaaldehydate	1931-63-1	1434	1439	0.04	0.35	0.27	0.11	0.06	0.04	<0.01
	Methyl dodecanoate	111-82-0	1524	1522	1.25	0.28	0.22	0.12	0.12	0.08	<0.01
	Methyl myristoleate	56219-06-8	1714	1715	1.82	0.00	0.00	0.00	0.00	0.00	<0.01
	Methyl tetradecanoate	124-10-7	1726	1724	10.01	3.21	2.72	1.17	1.14	0.79	<0.01
	Methyl pentadecanoate	7132-64-1	1826	1820	1.93	0.74	0.72	0.47	0.52	0.29	<0.01
	Methyl palmitoleate	1120-25-8	1907	1899	3.82	1.44	1.22	0.93	0.77	0.79	<0.01
	Methyl hexadecanoate	112-39-0	1927	1927	16.48	10.75	10.52	7.87	6.70	5.27	<0.01
Alkanes	Octane	111-65-9	802	801	0.00	1.37	1.45	2.26	2.30	1.62	<0.01
	Pentane	109-66-0	502	500	0.19	0.39	0.32	0.43	0.43	0.38	<0.01
	Hexane	110-54-3	602	600	0.20	0.67	0.48	0.72	0.73	0.67	ns
	Heptane	142-82-5	701	700	3.32	2.73	2.06	2.99	3.41	3.38	<0.01
	Undecane	1120-21-4	1101	1100	0.25	0.17	0.13	0.30	0.21	0.24	ns
	Tridecane	629-50-5	1302	1300	0.13	0.22	0.23	0.28	0.25	0.27	ns
	Tetradecane	629-59-4	1402	1400	0.16	0.33	0.32	0.37	0.40	0.39	ns
	Pentadecane	629-62-9	1502	1500	0.33	0.46	0.53	0.54	0.60	0.64	ns
	Hexadecane	544-76-3	1601	1600	0.24	0.35	0.32	0.33	0.47	0.47	ns
	5-Phenyl undecane	4537-15-9	1640	1632	0.08	0.10	0.09	0.07	0.13	0.13	ns
	4-Phenyl undecane	4536-86-1	1652	1643	0.04	0.09	0.07	0.05	0.12	0.11	ns
	Heptadecane	629-78-7	1701	1700	0.63	0.69	0.71	0.70	1.05	1.09	ns
	6-Phenyl dodecane	2719-62-2	1735	1726	0.03	0.03	0.04	0.03	0.06	0.06	ns
	3-Phenyl dodecane	2400-00-2	1779	1766	0.10	0.21	0.10	0.08	0.14	0.13	ns
	Octadecane	593-45-3	1799	1810	2.22	3.31	0.89	0.65	1.77	3.45	ns
Acids	Acetic acid	64-19-7	577	559	1.44	1.51	1.65	1.37	1.27	1.38	ns
	Pentanoic acid	109-52-4	881	868	0.36	0.36	0.43	0.43	0.46	0.41	ns
	Hexanoic acid	142-62-1	968	983	0.62	0.49	0.56	0.54	0.51	0.61	ns
	Heptanoic acid	111-14-8	1066	1080	0.58	0.68	0.80	0.83	0.96	0.91	ns
	Octanoic acid	124-07-2	1164	1160	0.71	1.45	1.27	1.23	0.95	0.99	ns
	Nonanoic acid	112-05-0	1264	1276	1.85	1.97	2.26	2.33	2.67	2.74	ns
	Decanoic acid	334-48-5	1359	1358	0.95	1.03	1.39	1.24	1.44	1.33	ns
	Dodecanoic acid	143-07-7	1557	1555	0.52	0.58	0.66	0.62	0.84	0.73	ns
	Tetradecanoic acid	544-63-8	1757	1760	0.65	0.60	0.82	0.75	1.21	1.05	ns
	Hexadecanoic acid	57-10-3	1967	1967	3.42	3.34	3.73	4.09	5.07	5.37	ns
Benzenes	Benzene	71-43-2	662	669	1.03	0.73	1.15	0.71	0.43	0.42	ns
	Toluene	108-88-3	770	763	0.54	0.37	0.49	0.51	0.32	0.32	ns
	Ethylbenzene	100-41-4	866	867	0.13	0.14	0.14	0.11	0.11	0.08	ns
	p-Xylene	106-42-3	868	863	0.23	0.21	0.26	0.20	0.20	0.19	ns
	o-Xylene	95-47-6	899	908	0.23	0.22	0.26	0.20	0.20	0.20	ns
	Benzophenone	119-61-9	1656	1653	0.11	0.17	0.19	0.16	0.23	0.20	ns
	Indole	120-72-9	1306	1296	0.13	0.13	0.15	0.11	0.17	0.17	ns
Ketones	2-Butanone	78-93-3	599	597	0.49	0.44	1.07	0.97	0.68	0.79	ns
	2-Pentanone	107-87-9	684	687	0.84	0.95	1.24	1.05	0.98	1.12	ns
	3-Heptanone	106-35-4	886	887	0.10	0.25	0.22	0.20	0.13	0.20	ns
	4-Octanone	589-63-9	974	977	0.40	0.41	0.45	0.42	0.38	0.40	ns
	Acetophenone	98-86-2	1074	1079	0.07	0.10	0.13	0.10	0.11	0.10	ns
	2-Nonanone	821-55-6	1094	1094	0.17	0.28	0.28	0.26	0.29	0.29	ns
Alcohols	1-Hexanol	111-27-3	867	868	0.05	0.24	0.33	1.28	0.25	0.16	ns
	1-Octanol	111-87-5	1071	1071	0.26	1.29	1.17	1.22	1.09	0.92	<0.01
	1-Tetradecanol	112-72-1	1681	1671	0.10	0.25	0.25	0.24	0.25	0.28	ns
Furans	2-Furanmethanol	98-00-0	852	850	0.09	0.10	0.12	0.08	0.12	0.07	ns
	2-Pentylfuran	3777-69-3	993	991	0.28	1.70	1.33	1.51	1.19	1.19	<0.01
	2-Octylfuran	4179-38-8	1296	1286	0.02	0.08	0.06	0.07	0.07	0.08	<0.01
Pyrazines	2,6-Dimethylpyrazine	108-50-9	918	912	0.00	0.00	0.00	0.02	0.03	0.07	<0.01
	3-Ethyl-2,5-dimethylpyrazine	13360-65-1	1080	1072	0.00	0.00	0.00	0.00	0.00	0.13	<0.01
	2,5-Dimethyl-3-isoamylpyrazine	111150-30-2	1319	1322	0.00	0.00	0.00	0.00	0.12	0.30	<0.01
Lactones	y-crotonolactone	497-23-4	912	916	0.38	0.37	0.32	0.31	0.39	0.34	ns
	δ-Tetradecalactone	2721-22-4	1939	1938	2.74	3.67	3.71	3.98	4.16	4.95	ns
Alkenes	1-Octene	111-66-0	792	791	0.18	0.20	0.40	0.57	0.69	0.68	ns
Amines	Trimethylamine	75-50-3	480	479	1.18	1.16	1.01	1.19	1.30	1.19	0.02
Terpenes	Neophytadiene	504-96-1	1841	1837	1.32	1.02	0.64	0.83	2.52	2.70	ns
Total					100.00	100.00	100.00	100.00	100.00	100.00	

VOCs: Volatile organic compounds. CAS: Chemical abstract service. LRI: Linear retention index. Ref LRI: Referenced linear retention index from the literature/online resources. ns: Not significantly different. R (raw), G55 (very-rare), G60 (rare), G71 (medium-rare), G77 (well-done), and G85 (very well-done).

**Table 2:** Odour descriptors and odour thresholds of volatile compounds in raw and grilled beef with a VIP score > 1.

Individual VOCs	CAS Number	VIP Score	Odour Descriptor	Odour Ref	Odour Threshold * (ppm)	Odour Threshold Ref
Methyl heptanoate	106-73-0	2.244	Faint, waxy, nearly tasteless	[50]	0.004	[51]
Methyl hexanoate	106-70-7	2.235	Ethereal fruity, pineapple-like	[50]	0.039–0.43	[51]
Methyl octanoate	111-11-5	2.099	Strong, winey, fruity, orange-like	[52]	0.27–0.87	[51]
2,5-Dimethyl-3-isoamylpyrazine	111150-30-2	1.720	Sweet, fragrant	[53]	na	
2,6-Dimethylpyrazine	108-50-9	1.709	Coffee, nutty	[54]	1.5	[51]
Methyl butanoate	623-42-7	1.681	Ethereal fruity-apple odour, apple-like taste	[50]	0.043	[51]
Methyl 3-methylbutanoate	556-24-1	1.620	Strong, fruity, ethereal, pineapple-apple	[50]	0.0044–0.044	[51]
Methyl nonanoate	1731-84-6	1.607	Coconut	[55]	0.0005	[51]
Methyl hexadecanoate	112-39-0	1.544	Green, fruity, fatty	[56]	>2	[51]
Methyl 2-methylbutanoate	868-57-5	1.534	Fruity, sweet, apple, berry, ripe tropical notes	[50]	0.0001–0.00014	[51]
Methyl decanoate	110-42-9	1.502	wine	[55]	0.0043–0.0088	[51]
Benzaldehyde	100-52-7	1.496	Volatile almond oil, bitter almond, burning aromatic taste	[57]	0.35	[51]
Nonanal	124-19-6	1.473	Citrus-like, soapy	[58]	0.001	[51]
3-Ethyl-2,5-dimethyl-pyrazine	13360-65-1	1.403	Earthy, musty	[53]	0.0004	[51]
Methyl tetradecanoate	124-10-7	1.403	Orris	[59]	na	
Methyl palmitoleate	1120-25-8	1.396	na		na	
Heptanal	111-71-7	1.385	Oily, fatty, rancid, unpleasant, penetrating fruity odour in liquid	[57]	0.003	[51]
Octanal	124-13-0	1.342	Citrus-like, green	[58]	0.007	[51]
Methyl dodecanoate	111-82-0	1.303	Coconut, fatty	[59]	na	
Methyl pentadecanoate	7132-64-1	1.214	na		na	
(E)-2-Nonenal	18829-56-6	1.212	Fatty, green	[58]	0.00008	[51]
Trimethylamine	75-50-3	1.199	Fishy	[60]	0.0005	[51]
Hexanal	66-25-1	1.187	Green, grassy	[58]	0.005	[51]
Pentane	109-66-0	1.176	Very slight warmed-over flavour, oxidized	[57]	na	[51]
Decanal	112-31-2	1.162	Orange, citrus	[52]	0.003	[51]
Methyl myristoleate	56219-06-8	1.135	Geranium, metallic, pungent	[59]	na	
Methyl azelaaldehydate	1931-63-1	1.115	na		na	
Octane	111-65-9	1.109	Hydrocarbon odour (weak)	[50]	10	[51]
Methyl propionate	554-12-1	1.107	Reminiscent of rum	[52]	0.1	[51]
(E,E)-2,4-Decadienal	25152-84-5	1.036	Fatty, deep fried	[58]	0.00007	[51]

VOCs: Volatile organic compound. VIP: Variable Importance In Projection. CAS: Chemical Abstract Service. Ref: Reference. * Odour threshold in water. ppm: parts per million. na: Not available.

## Data Availability

The original contributions presented in this study are included in the article/Supplementary Material. Further inquiries can be directed to the corresponding author.

## Supplementary Materials

The following supporting information can be downloaded at https://www.mdpi.com/article/10.3390/foods14244239/s1; Table S1: Percentage of chemical classes in raw and grilled beef.

## Abbreviations

The following abbreviations are used in this manuscript:

VOC	Volatile organic compounds
GC–MS	Gas chromatography–mass spectrometry
DI-HiSorb	Direct immersion high-capacity sorptive extraction
HS-SPME	Headspace solid-phase microextraction
