# Supplementary material for: Impact of Different Grilling Temperatures on the Volatile Profile of Beef
Source: Foods. 2025 Dec 10;14(24):4239. doi: 10.3390/foods14244239 (PMC12732222; doi:10.3390/foods14244239)
Supplement: Supplementary file 1 [file foods-14-04239-s001.zip › foods-3985354-supplementary.pdf]

Supplementary Table S1. Percentage of Chemical Classes in Raw and Grilled Beef.

Chemical Class	Abundances %						
	**R**	**G55**	**G60**	**G71**	**G77**	**G85**	**p-value**
**Esters**	53.5	21.9	21.0	13.9	12.1	10.2	< 0.01
**Aldehydes**	16.4	40.4	42.3	46.6	44.0	43.8	< 0.01
**Pyrazines**	0.0	0.0	0.0	0.0	0.1	0.5	< 0.01
**Alcohols**	0.4	1.8	1.8	2.7	1.6	1.4	< 0.01
**Furans**	0.4	1.9	1.5	1.7	1.4	1.3	< 0.01
**Alkenes**	0.2	0.2	0.4	0.6	0.7	0.7	0.02
**Terpenes**	1.3	1.0	0.6	0.8	2.5	2.7	0.03
**Benzenes**	2.4	2.0	2.6	2.0	1.7	1.6	*ns*
**Alkanes**	7.9	11.1	7.7	9.8	12.1	13.0	*ns*
**Amines**	1.2	1.2	1.0	1.2	1.3	1.2	*ns*
**Lactones**	3.1	4.0	4.0	4.3	4.6	5.3	*ns*
**Ketones**	2.1	2.4	3.4	3.0	2.6	2.9	*ns*
**Acids**	11.1	12.0	13.6	13.4	15.4	15.5	*ns*
**Total**	100.0	99.9	99.9	100.0	100.1	100.1	

R (raw), G55 (very-rare), G60 (rare), G71 (medium-rare), G77 (well-done), G85 (very well-done)

ns: not significant
